# Analysis of APPL1 Gene Polymorphisms in Patients with a Phenotype of Maturity Onset Diabetes of the Young

**DOI:** 10.3390/jpm10030100

**Published:** 2020-08-25

**Authors:** Dinara E. Ivanoshchuk, Elena V. Shakhtshneider, Oksana D. Rymar, Alla K. Ovsyannikova, Svetlana V. Mikhailova, Pavel S. Orlov, Yuliya I. Ragino, Mikhail I. Voevoda

**Affiliations:** 1 Federal research center Institute of Cytology and Genetics, Siberian Branch of Russian Academy of Sciences (SB RAS), Prospekt Lavrentyeva 10, Novosibirsk 630090, Russia or shakhtshneyderev@bionet.nsc.ru (E.V.S.); mikhail@bionet.nsc.ru (S.V.M.); orlovpavel86@gmail.com (P.S.O.); mvoevoda@ya.ru (M.I.V.); 2 Institute of Internal and Preventive Medicine—Branch of Institute of Cytology and Genetics, SB RAS, Bogatkova Str. 175/1, Novosibirsk 630004, Russia; orymar23@gmail.com (O.D.R.); aknikolaeva@bk.ru (A.K.O.); ragino@mail.ru (Y.I.R.)

**Keywords:** maturity onset diabetes of the young type 14, diabetes mellitus, APPL1, single-nucleotide variant, population

## Abstract

The *APPL1* gene encodes a protein mediating the cross-talk between adiponectin and insulin signaling. Recently, it was found that *APPL1* mutations can cause maturity onset diabetes of the young, type 14. Here, an analysis of *APPL1* was performed in patients with a maturity-onset diabetes of the young (MODY) phenotype, and prevalence of these mutations was estimated in a Russian population, among type 2 diabetes mellitus (T2DM) and MODY patients. Whole-exome sequencing or targeted sequencing was performed on 151 probands with a MODY phenotype, with subsequent association analysis of one of identified variants, rs11544593, in a white population of Western Siberia (276 control subjects and 169 T2DM patients). Thirteen variants were found in *APPL1*, three of which (rs79282761, rs138485817, and rs11544593) are located in exons. There were no statistically significant differences in the frequencies of rs11544593 alleles and genotypes between T2DM patients and the general population. In the MODY group, AG rs11544593 genotype carriers were significantly more frequent (AG vs. AA + GG: odds ratio 1.83, confidence interval 1.15–2.90, *p* = 0.011) compared with the control group. An association of rs11544593 with blood glucose concentration was revealed in the MODY group. The genotyping data suggest that rs11544593 may contribute to carbohydrate metabolism disturbances.

## 1. Introduction

Timely verification of a diabetes mellitus type is important because aside from diabetes mellitus types 1 and 2 and gestational diabetes, there are rarer types of this disease. Maturity onset diabetes of the young (MODY) is an inherited type of autosomal dominant diabetes mellitus with early onset and primarily involves a reduction in β-cell function without autoantibodies. MODY differs from the main diabetes types—type 1 diabetes mellitus (T1DM) and T2DM—in the clinical course, treatment strategies, and prognosis [1].

To date, 14 types of MODY (MODY1 through MODY14) have been identified, each associated with mutations in a specific gene: *HNF4A*, *GCK*, *HNF1A*, *PDX1*, *HNF1B*, *NEUROD1*, *KLF11*, *CEL*, *PAX4*, *INS*, *BLK*, *KCNJ11*, *ABCC8*, and *APPL1* [2,3,4]. Eleven percent to 30% of MODY cases are caused by impairment of functions of other genes [2,3].

The *APPL1* gene (adaptor protein, phosphotyrosine-interacting with the PH domain and leucine zipper 1) is located in chromosomal region 3p14.3 and contains 23 exons. APPL1 is a multifunctional adaptor protein that consists of 710 amino acid residues (aa) and is characterized by five key functional domains: an NH_2_-terminal Bin/Amphiphysin/Rvs domain (BAR domain; aa 17–268, identified as a leucine zipper), a central pleckstrin homology domain (PH domain; aa 278–374), motif between PH and PTB domains (BPP domain, aa 375–499), a COOH-terminal phosphotyrosine-binding domain (PTB domain, aa 500–625), and a coiled-coil (CC domain, aa 625–710) [5,6]. All the domains of APPL1 can bind to lipids, and each domain has unique binding affinity [6].

In humans, high levels of *APPL1* expression have been found in the liver, adipose tissue, muscles, brain, and pancreas [6]. Chinese patients with newly diagnosed T2DM have elevated serum APPL1 levels [7].

APPL1 can interact with more than 50 different proteins regulating multiple signaling pathways depending on the cell type [8]. APPL1 binds to AKT2 (RAC-beta serine/threonine-protein kinase or AKT serine/threonine kinase 2), a key molecule in the insulin signaling pathway, thereby enhancing insulin-induced AKT2 activation and downstream signaling in the liver, skeletal muscle, adipocytes, and endothelium. In the liver, APPL1 potentiates the inhibitory effect of insulin on hepatic gluconeogenesis through activation of AKT protein kinases; APPL1 overexpression in the liver eliminates hyperglycemia in mice with diabetes. In skeletal muscle and adipocytes, APPL1 mediates insulin-stimulated glucose uptake by controlling the translocation of cytosolic glucose transporter 4 to the plasma membrane [5].

Additionally, APPL1 plays an important role in the regulation of insulin metabolism and insulin resistance, being an adaptor in the adiponectin signaling pathway. Adiponectin, a hormone secreted by white adipose tissue, has anti-inflammatory and antidiabetic effects, enhances insulin sensitivity, exerts various actions on fertility, and regulates the processes of sexual and general maturation, pregnancy, and lactation [9,10]. A decrease in adiponectin levels is associated with obesity and metabolic syndrome. The PTB domain of APPL1 directly interacts with the N-terminal intracellular region of adiponectin receptors: AdipoR1 and AdipoR2. In adiponectin signaling, APPL1 mediates fatty acid oxidation and glucose metabolism by activating AMPK (AMP-activated protein kinase) and p38 MAPK (P38 mitogen-activated protein kinases) [10]. It is reported that APPL1 protein isoforms APPL2 [11] and APPL1sv (which is encoded by a murine splice variant of *App1* mRNA [12]), apparently act as negative regulators of adiponectin signaling. In *Appl1* knockout mice, adipocyte differentiation is negatively affected, and lipolysis is enhanced in mature adipocytes; IN contrast, *APPL1* overexpression evidently does not influence these processes [13]. APPL1 mediates AMPK phosphorylation in response to adiponectin [14]. It affects the thermogenesis of brown adipocytes via an interaction with HDAC3 (Histone deacetylase 3); this phenomenon has promising implications for the treatment of obesity [15].

In a mouse model of T1DM, investigators have demonstrated a negative action of APPL1 on the regulation of inflammation and apoptosis in β-cells of the pancreas [16].

Furthermore, downregulation of *APPL1* expression by small interfering RNA reduces the synergistic effect of adiponectin on insulin-stimulated AKT phosphorylation. Hence, the APPL1-mediated cross-talk between insulin and adiponectin signaling pathways may be a critical mechanism underlying the insulin-sensitizing effect of adiponectin [6].

It has been shown that some variants of the gene have functional significance. An association of single-nucleotide polymorphisms rs3806622 and rs4640525 (of the *APPL1* gene) with a distribution of adipose tissue has been found among T2DM patients [17]. In that study, the G allele frequencies of rs3806622 and rs4640525 were significantly higher among the T2DM patients with greater waist circumference [17]. Additionally, rs4640525 is associated with increased risk of coronary heart disease in patients with T2DM [6,18] and the risk of nonalcoholic fatty liver disease in Chinese Han population [19].

Two rare mutations, rs869320673 (Leu552Ter) and rs796065047 (Asp94Asn), in the *APPL1* gene have been identified through whole-exome sequencing in two of 60 large families with high prevalence of diabetes not due to mutations in known MODY-associated genes (*HNF4A*, *GCK*, *HNF1A*, *PDX1*, *HNF1B*, and *NEUROD1*) [4]. The Leu552Ter mutation (rs869320673, introduction of a premature stop codon at aa position 552) causes a deletion of most of the PTB domain, thereby making APPL1 unable to bind to AKT and abrogates APPL1 protein expression in transfected HepG2 cells. In the above study, the substitution was found in all the 10 family members with diabetes or prediabetes. Most of the unaffected carriers of this substitution were younger than 38 years (the median age at diabetes diagnosis among the affected family members) [4]. Missense mutation Asp94Asn (rs796065047) was identified in three generations of a US family with MODY [4]. The mutation was found or inferred to be present in five of the seven family members with diabetes. The missense mutation affects the aspartic acid residue at position 94, which is located on the concave surface of the APPL1 BAR domain and is highly conserved among various species [20]. The Asp94Asn mutation significantly attenuates insulin-stimulated AKT2 and GSK3β phosphorylation. This mutation was not found in 1,639 unrelated individuals of European ancestry without diabetes mellitus and 2970 patients with T2DM [4].

The aims of our study were identification of potentially pathogenic variants in the *APPL1* gene in patients with early-onset diabetes mellitus corresponding to a MODY phenotype by whole-exome sequencing and estimation of their prevalence in a control Russian population and among patients with T2DM.

## 2. Materials and Methods

The study protocol was approved by the local Ethics Committee of the Institute of Internal and Preventive Medicine (a branch of the Institute of Cytology and Genetics, SB RAS, Novosibirsk, Russia), protocol number 7, 22.06.2008. Written informed consent to be examined and to participate in the study was obtained from each patient. For individuals younger than 18 years, the informed consent form was signed by a parent or legal guardian.

A group with a clinical diagnosis of MODY (MODY phenotype) consisted of 151 unrelated patients aged 8 to 35 years (26.1 ± 14.4 years (mean ± SD (standard deviation)); males: 47%). The inclusion criteria were as follows: a verified diagnosis of diabetes, a debut of the disease for probands at the age of 35 years and earlier, a family history of diabetes mellitus, the absence of obesity, the absence of antibodies against pancreas islet cells and glutamic acid decarboxylase, sufficient secretory function of β-cells, no need for insulin therapy, and the absence of ketoacidosis at the onset of the disease. The following exclusion criteria were applied: a history of tuberculosis or infection with the human immunodeficiency virus, an infectious disease caused by hepatitis B or C virus that requires antiviral treatment, and substance abuse or alcoholism for two years before the examination. The group with a MODY phenotype may have included patients with MODY, patients with T1DM and negative test results on antibodies, and patients with early-onset T2DM.

At first-time diagnosis of diabetes, most patients had no clinical symptoms of a carbohydrate metabolism disorder; hyperglycemia was diagnosed during routine examinations. In 33% of the females, diabetes manifested itself during pregnancy. In the patients with a MODY phenotype, glycated hemoglobin and C-peptide levels were close to normal. All the patients underwent continuous glucose monitoring for three days. The level of glycemia was 7.2 ± 2.7 mmol/L (mean ± SD); the minimum was 2.8 mmol/L, and maximum 12.8 mmol/L.

Blood samples were collected from the ulnar vein for biochemical analysis in the morning on an empty stomach. Lipid levels (cholesterol, triglycerides, and low-density and high-density lipoprotein cholesterols) and glucose concentration were determined on a biochemical analyzer KoneLab 300i (USA) with Thermo Fisher Scientific reagents (USA). Genomic DNA was isolated from venous-blood leukocytes by phenol–chloroform extraction [21]. Quality of the extracted DNA was assessed by means of a capillary electrophoresis system (Agilent 2100 Bioanalyzer; Agilent Technologies Inc., Santa Clara, California, United States).

At the first stage, clinical exome sequencing was performed for 40 randomly selected patients from the MODY group. Clinical exome sequencing was carried out on an Illumina HiSeq 1500 instrument (Illumina, San Diego, CA, USA). The enrichment and library preparation were performed using the SureSelectXT Human All Exon v.5 + UTRs Kit. Whole-exome libraries were prepared with the AmpliSeq Exome Kit (Thermo Fisher Scientific). For other patients (111 unrelated probands), we performed targeted sequencing. In the target panel, we included coding parts and adjacent splicing sites of MODY-associated genes, e.g., *APPL1*. To prepare the libraries, oligodeoxynucleotide probes and the KAPA HyperPlus Kit (Roche, Switzerland) were employed. Quality of the analyzed DNA and of the prepared libraries was evaluated by means of a capillary electrophoresis system, Agilent 2100 Bioanalyzer (Agilent Technologies Inc., USA). Analysis of a fully prepared library was conducted on the Illumina MiSeq platform (Illumina, San Diego, CA, USA).

The sequence reads were mapped to the reference human genome (GRCh37) via the Burrow–Wheeler Alignment tool (BWA v.0.7.17) [22]. Polymerase chain reaction (PCR)-generated duplicates were removed in the Picard Tools (https://broadinstitute.github.io/picard/). A search for single-nucleotide variants (SNVs) was conducted using the Genome Analysis Toolkit v.3.3 package by the procedure for local remapping of short insertions/deletions and recalibration of read quality [23]. The depth of coverage was 34× to 53×. SNVs with genotype quality scores <20 and coverage depth <10× were filtered out and excluded from further analysis. In sequenced groups, we filtered sequence variants if they were present in 10 or more variant reads with a quality score ≥30. Annotation of the SNVs was performed in the ANNOVAR (ANNOtate VARiation) software [24] using gnomAD [25], ClinVar [26], and HGMD (The Human Gene Mutation Database) [27], and literature data were taken into account too. Possible functional and significant effects of SNVs were assessed by means of PolyPhen-2 v.2.2.5 [28], SIFT (The scale-invariant feature transform) [29], PROVEAN (Protein Variation Effect Analyzer) [30], LIST (Local Identity and Shared Taxa) [31], and MutationTaster [32]. Identified SNVs of the *APPL1* gene were verified by Sanger sequencing.

At the second stage, segregation analysis was performed for two variants, rs138485817 and rs11544593, in probands’ families.

At the third stage, minor allele frequency and prevalence of genotypes of rs11544593 was analyzed in a white population of West Siberia, among T2DM patients, and among patients with a MODY phenotype.

The control group consisted of 276 random residents of Novosibirsk aged 45 to 69 years (54.2 ± 0.4 years; males: 49%). The T2DM group consisted of 169 patients aged 45 to 69 years (59.0 ± 6.7 years; males: 45%) with glucose levels more than 11.1 mmol/L, according to the criteria of the American Diabetes Association. Both groups were randomly selected from the population of 9360 people interviewed within the framework of the HAPIEE (Health, Alcohol and Psychosocial factors in Eastern Europe) project (Wellcome Trust, Great Britain) “Determinants of cardiovascular diseases in Eastern Europe.” An epidemiological survey of the population involved demographic and social data, smoking and alcohol abuse, a dietary survey, a history of chronic diseases, medication use, the Rose Angina Questionnaire, anthropometry, a threefold measurement of blood pressure, spirometry, ECG (Electrocardiogram) recording, and assessment of the blood glucose level, lipid profile, and other biochemical parameters [33]. Rs11544593 was genotyped by means of the TaqMan SNP assay (Biolink, Russia) and a StepOnePlus 7900HT Real-Time PCR System (Thermo Fisher Scientific, USA). Relative risk of MODY for a genotype or allele was calculated as an odds ratio (OR) via Fisher’s exact test and Pearson’s χ^2^ test. Differences were considered statistically significant at *p* < 0.05. Verification of the Hardy–Weinberg equilibrium was carried out by the χ^2^ method. Differences in means of the quantitative indicators between genotypes were calculated after adjustment for sex, age, and the body mass index (BMI) via the GLM (The generalized linear model) model of IBM SPSS Statistics 23.0.

## 3. Results and Discussion

Pathogenic sequence variants in MODY-associated genes were found in 37 probands out of the 151 persons examined by whole-exome sequencing or targeted sequencing of the panel of genomic loci. Discovered variants segregated with a pathological phenotype in the families of the probands. Among the 37 cases, 24 pathogenic variants were detected in the *GCK* gene, 11 variants in *HNF1A*, one variant in the *ABCC8* gene, and one in *HNF1b*.

In the *APPL1* gene, 13 SNVs were found. Rs79282761, rs11544593, and rs138485817 are located in the coding part of the gene (Table 1).

In one case, synonymous substitution rs79282761 (Thr12Thr) was found. Missense substitution rs11544593 (Glu700Gly) in exon 22 was detected in 49 patients; six of them carried a pathogenic mutation in the *GCK* gene, another one in the *HNF1A* gene, and 42 patients had no mutations in MODY1–13 genes. Rs138485817 (Ser673Cys) was found in one patient without mutations in other MODY-associated genes. In silico analysis of rs11544593 and rs138485817 predicted their potential damaging effect according to testing in PolyPhen-2 (score: 0.455 and 0.999), SIFT (score: 0.007 and 0.02), and LIST (score: 0.828 and 0.902), respectively. The PROVEAN software estimated these substitutions as neutral (score: −1.55 and −0.603). MutationTaster predicted rs11544593 to be neutral (score: 98) and rs138485817 to be disease causing (score: 112).

The Glu700Gly (rs11544593) substitution and Ser673Cys (rs138485817) are located in the C-C domain of APPL1; this domain is responsible for direct binding of this protein to insulin receptor β subunit (IRβ), TrkA receptor tyrosine kinase, and the GIPC1 protein [34]. Nevertheless, the identified variants did not segregate with the pathological phenotype in the families of the probands.

We assessed population prevalence and association with biochemical parameters for rs11544593 (as a more prevalent variant) in the control population, among T2DM patients, and among the patients with a MODY phenotype (Table 2).

The general-population group was in Hardy–Weinberg equilibrium for rs11544593: χ^2^ = 3.05. There were no significant differences in the prevalence rates of genotypes and alleles of the rs11544593 polymorphism between T2DM patients and the general-population group (Table 2).

Significantly lower prevalence of the A allele (OR = 1.57, 95% confidence interval (CI) 1.06–2.32, *p* = 0.03) was observed among the patients with a MODY phenotype (Table 2) compared with the general population. The frequency of the less common G allele of rs11544593 (in the *APPL1* gene) was 0.17 in group MODY and 0.13 in group T2DM. In the general-population group, G allele frequency was 0.12, in agreement with the gnomAD database (0.13). Thus, we found that in the Russian population, G allele frequency of rs11544593 is higher in the presence of a MODY phenotype, which is characterized by early onset of carbohydrate metabolism disorders (Table 2). In the MODY group, AG genotype carriers were significantly more frequent (AG vs. AA + GG: OR = 1.83, 95% CI 1.15–2.90, *p* = 0.011) and carriers of the AA genotype were less common (AA vs. AG + GG: OR = 0.57, 95% CI 0.36–0.89, *p* = 0.014) as compared with the general population (Table 2). For further investigation of the observed effect of heterozygous-variant carriage on the phenotype, a bigger sample size is needed.

rs11544593 with the blood glucose level was revealed in the patients with a MODY phenotype. The highest average values of blood glucose levels were detected in the MODY patients with the GG genotype of rs11544593 (*p* = 0.004; Table 3). There was no association of rs11544593 with the blood glucose level among the patients with T2DM and in the control group.

We confirmed that mutations in the *APPL1* gene, depending on their location, may cause either autosomal dominant diabetes of the MODY14 type [4] or milder aberrations of blood glucose concentration.

There were no statistically significant associations of rs11544593 with sex, immunoreactive insulin, C-peptide, cholesterol, triglycerides, low-density and high-density lipoprotein cholesterols, triglyceride levels, BMI, atherogenicity index, waist circumference, and the waist/hip ratio.

Of note, there are some data on an association of rs11544593 with the BMI in a white American population consisting partly of individuals with T2DM [35]. We did not find an association of rs11544593 with either the BMI or T2DM. Apparently, this polymorphism can influence glucose and/or fatty acid metabolism, but its penetrance is incomplete. Although APPL1 is known to be an adaptor in multiple signaling pathways, phenotypic manifestations of its variants remain elusive. It is known that adiponectin has an antiatherosclerotic effect [36]. In atherosclerotic plaques of patients with T2DM, APPL1 is underexpressed relative to patients without diabetes [36]. In the latter study, in the group of patients with diabetes, subjects taking incretins showed higher APPL1 and adiponectin levels than did subjects who had never taken incretins [36]. This finding is interesting in terms of research into the association of *APPL1* variants with lipid metabolism disorders among patients with diabetes.

## 4. Conclusions

For the first time, a genetic analysis of the *APPL1* gene was performed on Russian patients with early-onset diabetes mellitus that phenotypically corresponds to MODY. Our whole-exome sequencing did not reveal novel or previously described rare pathogenic substitutions (Leu552Ter and Asp94Asn) in the *APPL1* gene that are associated with MODY14. The genotyping results and glucose level data obtained from the general-population group and patients with carbohydrate metabolism disorders suggest that rs11544593 (located in the *APPL1* gene) may contribute to earlier onset of carbohydrate metabolism disorders. Further research on rs11544593 in a larger study population and identification of polymorphisms of the genes encoding binding partners of APPL1 in signaling cascades hold promise for elucidation of the pathogenesis of diabetes phenotypes. The present study has some limitations. We examined only rs11544593 and traditional diabetes risk factors and thus could not rule out the influence of other factors, such as lifestyle factors and other genetic factors that may affect the results of observational studies. In some cases, MODY is caused by de novo mutations lacking a family history. In these situations, a clinician should focus on the specific clinical features of the patient’s diabetes that are different from the typical course of T1DM and T2DM [37].

## Figures and Tables

**Table 1:** The *APPL1* gene variants among the patients with a maturity-onset diabetes of the young phenotype in Russia, and the SNVs’ (single nucleotide variants) minor allele frequency according to the gnomAD database.

Name of an SNV.	Location	Substitution (NM_012096.3)	Minor Allele Frequency (gnomAD)
rs113307246	5′UTR	c.-151=	0.005
rs79282761	Exon 1	Thr12Thr c.36G>C	C = 0.01
rs6789847	Intron 1	c.54+2907T>A	T = 0.07
rs200584055	Intron 12	c.1096-30del	delA = 0.03
rs62251992	Intron 17	c.1484-71A>G	G = 0.12
rs10510791	Intron 17	c.1658+38C>G	G = 0.46
rs1533272	Intron 18	c.1695+114T>A	C = 0.38
rs11544593	Exon 22	Glu700Gly c.2099A>G	G = 0.13
rs138485817	Exon 22	Ser673Cys c.2018 C>G	G = 0.0006
rs1046545	3′UTR	c.*1455=	T = 0.19
rs3204124	3′UTR	c.*1528=	G = 0.68
rs3087684	3′UTR	c.*2604=	C = 0.68
rs1913302	3′UTR	c.*3246=	A = 0.68

* Stop codon.

**Table 2:** Frequencies of alleles and genotypes of rs11544593 in the general-population group (Western Siberia; control), in the type 2 diabetes mellitus group, and among patients with a maturity-onset diabetes of the young phenotype in Russia.

	MODY (*n* = 151)	T2DM (*n* = 169)	General Population (*n* = 276)	OR (An Odds Ratio), 95% CI (A Confidence Interval)	*p*
Genotypes					
AA	67.55 (102)	76.33 (129)	78.62 (217)	1 vs. 3, 0.57 (0.36–0.89)	0.014
				2 vs. 3, 0.88 (0.56–1.38)	>0.05
AG	29.80 (45)	21.30 (36)	18.84 (52)	1 vs. 3, 1.83 (1.15–2.90)	0.011
				2 vs. 3, 1.17 (0.72–1.88)	>0.05
GG	2.65 (4)	2.37 (4)	2.54 (7)	1 vs. 3, 1.05 (0.30–3.63)	>0.05
				2 vs. 3, 0.93 (0.27–3.23)	>0.05
**Alleles**					
A	82.3	86.98	88.04	1 vs. 3, 1.57 (1.06–2.32)	0.03
G	17.7	13.02	11.96	2 vs. 3, 0.91 (0.60–1.37)	>0.05

**Table 3:** Mean blood glucose levels (mM/L).

rs11544593 Genotypes	MODY Phenotype Patients X (S_x_)	T2DM Patients X (S_x_)	General Population X (S_x_)
AA	6.9 (0.2)	11.9 (0.4)	5.2 (0.1)
AG	6.0 (0.3)	12.2 (0.8)	5.8 (0.2)
GG	8.7 (0.8)	14.5 (2.)	5.4 (0.6)
Age (p)	0.000 *	0.508	0.508
Sex (p)	0.422	0.334	0.379
Genotype (p)	0.004 *	0.523	0.050

Continuous variables are presented as mean (X) ± standard error (S_x_). * A statistically significant difference from the MODY group and general population.
